# EEG Delta/Theta Ratio and Microstate Analysis Originating Novel Biomarkers for Malnutrition-Inflammation Complex Syndrome in ESRD Patients

**DOI:** 10.3389/fnhum.2021.795237

**Published:** 2022-01-04

**Authors:** Tirapoot Jatupornpoonsub, Paramat Thimachai, Ouppatham Supasyndh, Yodchanan Wongsawat

**Affiliations:** ^1^Brain-Computer Interface Laboratory, Department of Biomedical Engineering, Faculty of Engineering, Mahidol University, Salaya, Thailand; ^2^Division of Nephrology, Department of Medicine, Phramongkutklao Hospital, Bangkok, Thailand

**Keywords:** delta/theta ratio, microstate analysis, end-stage renal disease, malnutrition-inflammation complex syndrome, encephalopathy, cognitive decline, EEG biomarker

## Abstract

The Malnutrition-Inflammation Score (MIS) was initially proposed to evaluate malnutrition-inflammation complex syndrome (MICS) in end-stage renal disease (ESRD) patients. Although MICS should be routinely evaluated to reduce the hospitalization and mortality rate of ESRD patients, the inconvenience of the MIS might limit its use. Cerebral complications in ESRD, possibly induced by MICS, were previously assessed by using spectral electroencephalography (EEG) via the delta/theta ratio and microstate analysis. Correspondingly, EEG could be used to directly assess MICS in ESRD patients, but the relationships among MICS and these EEG features remain inconclusive. Thus, we aimed to investigate the delta/theta ratio and microstates in ESRD patients with high and low risks of MICS. We also attempted to identify the correlation among the MIS, delta/theta ratio, and microstate parameters, which might clarify their relationships. To achieve these objectives, a total of forty-six ESRD subjects were willingly recruited. We collected their blood samples, MIS, and EEGs after receiving written informed consent. Sixteen women and seven men were allocated to low risk group (MIS ≤ 5, age 57.57 ± 14.88 years). Additionally, high risk group contains 15 women and 8 men (MIS > 5, age 59.13 ± 11.77 years). Here, we discovered that delta/theta ratio (*p* < 0.041) and most microstate parameters (*p* < 0.001) were significantly different between subject groups. We also found that the delta/theta ratio was not correlated with MIS but was strongly with the average microstate duration (ρ = 0.708, *p* < 0.001); hence, we suggested that the average microstate duration might serve as an alternative encephalopathy biomarker. Coincidentally, we noticed positive correlations for most parameters of microstates A and B (0.54 ≤ ρ ≤ 0.68, *p* < 0.001) and stronger negative correlations for all microstate C parameters (−0.75 ≤ ρ ≤ −0.61, *p* < 0.001). These findings unveiled a novel EEG biomarker, the MIC index, that could efficiently distinguish ESRD patients at high and low risk of MICS when utilized as a feature in a binary logistic regression model (accuracy of train-test split validation = 1.00). We expected that the average microstate duration and MIC index might potentially contribute to monitor ESRD patients in the future.

## 1. Introduction

Malnutrition-inflammation complex syndrome (MICS), a condition in which protein-energy malnutrition (PEM) and inflammation are present simultaneously, has been known to particularly occur in end-stage renal disease (ESRD) patients, leading to a high rate of hospitalization and mortality (Kalantar-Zadeh et al., 2003). The point prevalence of MICS in dialysis patients has been reported as 42% to 62% in the previous literature (Bramania et al., 2020). It was also suggested that nutritional supplement, optimized dialysis, and anti-inflammatory interventions may possibly alleviate MICS in these patients (Kalantar-Zadeh et al., 2003). To diagnose MICS, scoring systems that combined the evaluation of both nutritional status and inflammation were ideally recommended to utilize (Kalantar-Zadeh et al., 2003). The Malnutrition-Inflammation Score (MIS) was previously introduced as a comprehensive and reliable questionnaire for evaluating the severity of MICS. Studies have indicated that the MIS can also assess the mortality and morbidity of ESRD patients (Kalantar-Zadeh et al., 2001; Ho et al., 2008; Harvinder et al., 2016), in which a cutoff value of five indicates 88% certainty of malnutrition and an 80% risk of 1-year mortality in ESRD patients (Ho et al., 2008; Harvinder et al., 2016). Although the MICS status of an ESRD patient needs to be routinely monitored to prevent undesirable outcome, the MIS questionnaire is invasive and time-consuming, since it requires the laboratory serum analysis and a clinician to evaluate the patient properly. Hence, there may be an opportunity to improve the assessment of MICS in ESRD patients.

Another clinical consideration for ESRD patients is that more than 80% may present neurological complications (Hamed, 2019). In fact, MICS may induce cerebral complications, including cognitive deterioration and encephalopathy (Kalantar-Zadeh et al., 2003; Zheng et al., 2017; Guenzani et al., 2019; Hamed, 2019; Jatupornpoonsub et al., 2021). Spectral analysis of electroencephalogram (EEG), a non-invasive and feasible neuroimaging tool, has been utilized to assess these cerebral complications in prior studies. Although an obvious mechanism of delta/theta ratio deviation in encephalopathy is still inconclusive, researchers have found that the delta/theta ratio tended to decrease (increased theta/delta ratio) in encephalopathy without specificity of the underlying causes (Faigle et al., 2013; Kamiya-Matsuoka and Tummala, 2017; Jatupornpoonsub et al., 2021). It was also suggested that delta/theta ratio inversely reflects the severity of encephalopathy in ESRD patients (Jatupornpoonsub et al., 2021). It is possible that this ratio may be altered by the combination of encephalopathy consequences, which affected both delta and theta oscillation. In details, the variation of these activities has been found to associate with short attention span and mild cognitive decline, which were found to be signs of early encephalopathy in ESRD patients (Kalantar-Zadeh et al., 2003; Hamed, 2019; Jatupornpoonsub et al., 2021). Therefore, the delta/theta ratio, may be particularly altered by the induction of MICS in ESRD patients.

Spectral analysis explicitly bases on frequency-domain EEG; on the other hand, microstate analysis establishes the assessment of quasistable patterns in time-domain EEG (Lehmann et al., 1987; Poulsen et al., 2018). In the prior study, different microstates have been said to involve different large-scale activities of a brain (Poulsen et al., 2018), each topographic pattern reflects a configuration of neuronal generators in an underlying neural network (Britz et al., 2010; Khanna et al., 2015). Following the argument that four microstates are consistently generated at rest, and each of which synchronously correlates with an activation of a resting state network (Britz et al., 2010), it may be reasonable to include four microstates in the resting state analysis of EEG. Specifically, microstate A, B, C, and D have been suggested to indicate resting activities in phonological processing network, visual processing network, salience network, and dorsal attention network (DANs), respectively (Mantini et al., 2007; Britz et al., 2010; Nishida et al., 2013; Khanna et al., 2015). In microstate analysis, an amount of each neural network activity is commonly reflected by microstate parameters including occurrence, coverage, and duration (Khanna et al., 2015; Michel and Koenig, 2018). These parameters have been found to randomly alter in many diseases, such as schizophrenia, dementia, cognitive decline, Alzheimer's disease, and head injury (Nishida et al., 2013; Khanna et al., 2015; Michel and Koenig, 2018; Musaeus et al., 2019, 2020). Therefore, it may be possible to discover an alteration of microstate pattern or parameters in ESRD patients with the MICS comorbidity.

Although EEGs could be applied to alternatively observe MICS in ESRD patients, the relationships among the MIS, the delta/theta ratio, and EEG microstate parameters remain unanswered. Therefore, we aimed to investigate the delta/theta ratio and microstates in ESRD patients with high (ESRD-H) and low (ESRD-L) risks of MICS, which are allocated using an MIS cutoff of five. Moreover, we attempted to identify the correlations among the MIS, delta/theta ratio, and microstate parameters, which might clarify their relationships. In the first investigation, we utilized quantitative EEG (QEEG) to calculate the delta/theta ratio compared to a normative database (Khanna et al., 2015; ANI, 2018). In the second investigation, we used microstate analysis to observe quasistable patterns and their parameters. We expected that the delta/theta ratio would be decreased in ESRD-H relative to ESRD-L because MICS should lead to more severe encephalopathy and that this ratio might be correlated with the MIS. Moreover, if the delta/theta ratio was correlated with some of the microstate parameters, it would indicate that these parameters might be related to encephalopathy. We also hypothesized that the microstate parameters of ESRD-H patients should be different from those of ESRD-L patients due to the effect of MICS on the underlying cerebral network. If the correlations between the MIS and either the microstate parameters or the delta/theta ratio were significantly strong, we would suggest an optimal biomarker for evaluating MICS in ESRD patients further.

## 2. Materials and Methods

### 2.1. Participants

The experimental protocol involving participants in this cross-sectional study was approved by the Institutional Review Board of Phramongkutklao Hospital with certificate of approval (COA) number S072h/62 and by the Institutional Review Board of Mahidol University with COA number MU-CIRB 2020/393.2511. ESRD patients who underwent either peritoneal dialysis (PD) or machine hemodialysis (MHD) were willingly recruited. They were asked to provide written informed consent before enrollment. All participants achieved sufficient dialysis; 3-4 times a week for MHD patients and dialysate drainage every 4 h for PD patients. The patients were aged at least 40 years and had no history of neurologic or psychiatric disease that could affect the EEG. Participants would be excluded if their EEGs contained excessive artifacts or if they recently received or had a history of exposure to central nervous system drugs, such as anti-epileptic and antidepressant drugs.

### 2.2. MICS Assessment

In this study, we used MIS questionnaires to assess MICS status of participants. This tool contains four sections: medical history, physical examination, body mass index (BMI), and blood test. The medical history section includes (1) dry weight alterations after dialysis for 3–6 months; (2) quantity of dietary intake; (3) gastrointestinal symptoms; (4) daily functional capacity; and (5) major comorbid conditions, including duration (years) of dialysis. The physical examination section consists of (6) loss of subcutaneous fat and (7) muscle wasting. The last two sections are (8) BMI and laboratory tests, including (9) serum albumin and (10) serum total iron binding capacity (TIBC) levels. Each component has four severity levels, which are scored from 0 to 3. The total score of MIS directly indicates the severity of MICS (Kalantar-Zadeh et al., 2001; Ho et al., 2008; Harvinder et al., 2016). Although the MIS can range from 0 to 30, the recommended cutoff between a low and high risk of MICS is five, which indicates an 88% probability of malnutrition in ESRD patients (Harvinder et al., 2016), as well as an 80% risk of 1-year mortality in these patients (Ho et al., 2008). Therefore, our study allocated participants to two subgroups: ESRD-H (MIS > 5) and ESRD-L (MIS ≤ 5). On the day before the participants were to undergo hemodialysis (for MHD) or 3 h after the last dialysate drainage (for PD), we collected their blood samples and MIS. Sex, age, and dialysis duration were matched to reduce comparison bias.

### 2.3. EEG Recording

After we had assessed the nutritional status of the participants, we asked them to undergo an eyes-closed EEG recording session for 6 min on the same day. Due to the COVID-19 pandemic, the participants needed to complete the recording while wearing a surgical mask. To obtain neutral resting-state EEG data, we made the experimental room soundproof and painted the walls ivory. The room was temperature controlled at 25 degrees Celsius and illuminated with sufficient light (300 lux). Before the session was started, we had requested that the participants sit upright and relaxed in an ergonomic chair with both legs forward in the most comfortable position. After that, the participants rested 1 min in the chair and were asked to close their eyes for a 5-min recording.

Microstate analysis has been said to still be reliable with a low electrode density recording (a minimum of eight electrodes) (Khanna et al., 2014). However, in practice, a higher electrode density might be more ideal but would lead to longer participant preparation and electrode attachment duration. Therefore, we decided to utilize the international 10-20 electrode placement system (19 channels) with reference to the left ear lobule (A1) and ground at the right ear lobule (A2) in this study. The referential EEG monopolar montage was measured under the following protocol to optimize the signal quality. We chose an elastic cap that was optimally fit to the participant's head, which ensured the closest distance between the titanium nitride electrodes and the scalp. After placing the cap, the gap between the electrodes and scalp was filled with conductive gel. Then, gold cup electrodes filled with conductive paste were attached to both ear lobules, and the impedance was maintained at less than 5 kΩ. Additionally, two cup electrodes were attached above the right eyebrow (positive electrode) and at the eyelid-cheek junction (reference) to obtain an electrooculogram (EOG) for assessing eye movement. Two more cup electrodes were placed at the left (positive electrode) and right (reference) wrists to record an electrocardiogram (ECG). These two signals were intended for use as EEG artifact-removal guides.

The EEG, EOG, and ECG signals were synchronously measured by a Brain Master Discovery 24E amplifier with a 256 Hz sampling rate and 24-bit accuracy. These signals were applied low-pass filter at 80 Hz, monitored, recorded, and stored in a laptop computer by using Brain Master Discovery software. Before recording, the signal offset was monitored on the acquisition screen and adjusted until it was less than 10 mV to maximize signal quality. During the recording period, eye blinks or muscle artifacts that affected the EEG were marked on the artifact record form, which was used to exclude contaminated EEG trials afterward.

### 2.4. Z-Score Power Ratio Calculation

The raw EEGs, recorded in the European Data Format (EDF), were processed, validated, and transformed using NeuroGuide software version 2.8.5 (ANI, 2018). Artifact-free EEG epochs were selected by using the EOG, ECG, and artifact-record forms as a guide. Test-retest and split-half reliability were then calculated to validate signal homogeneity and the consistency of a measurement as recommended by the manual (ANI, 2018). The selected EEGs were then downsampled to 128 Hz. A 5th-order Butterworth bandpass filter was applied with a passband of 1–40 Hz. The signals were converted to the frequency domain by fast Fourier transform (FFT) to calculate the absolute power and power ratio. In detail, absolute power is the diagonal of the auto-spectral matrix, which was calculated by the FFT of the signal multiplied by its complex conjugate and divided by the number of frequencies. The power ratio is the absolute power ratio of two specific bands (ANI, 2018). EEG frequency bands included in all calculations were specified as either delta (1.0–4.0 Hz) or theta (4.0–8.0 Hz), which were used to compute the delta/theta ratio. The power ratio was then transformed into a Z-score (in range −3.000 to 3.000) with reference to a normative database (Thatcher et al., 2003), in which patients were matched to healthy subjects by age, sex, and recording condition (eyes closed). Subsequently, the Z-scores were exported as tab delimiter text (TDT) files to undergo further statistical analysis.

### 2.5. Preprocessing Pipeline for Microstate Analysis

Python programming language version 3.8.10 was utilized to process and analyze the EEG signals. The open-source MNE-Python package version 0.23 was used to repair artifacts and perform microstate analysis on the EEG data (Gramfort et al., 2013). The raw EEG data in EDF were imported as input to an artifact repair pipeline. The raw signal was then subjected to a fourth-order Butterworth bandpass filter (1–40 Hz). The signal was prewhitened by scaling with the standard deviation across all channels. Then, the signal was decomposed with no dimensional reduction by using principal component analysis (PCA). The principal components were passed to the independent component analysis (ICA) algorithm, generating the independent components. Picard was chosen as the ICA algorithm rather than FastICA or Infomax, as Picard tends to be faster in terms of convergence and more robust to partially dependent sources, which is a common characteristic of EEG (Ablin et al., 2018). The EOG and ECG signals were matched to each independent component to calculate the Pearson correlation as an ICA score, which directly indicates any contaminated components. We also visualized this component by topographical plots, overlay plots, and property diagnostic plots. Then, we excluded outliers and reconstructed the optimized EEG signal. To handle artifacts such as theta bursts during drowsiness or other benign variants, we also manually selected the resting-state EEG in the time domain. The selected EEG was then saved in FIF format, which allows all descriptive information to be stored in the same file. The FIF file was then subjected to microstate analysis.

### 2.6. Microstate Analysis

**Figure 1:**
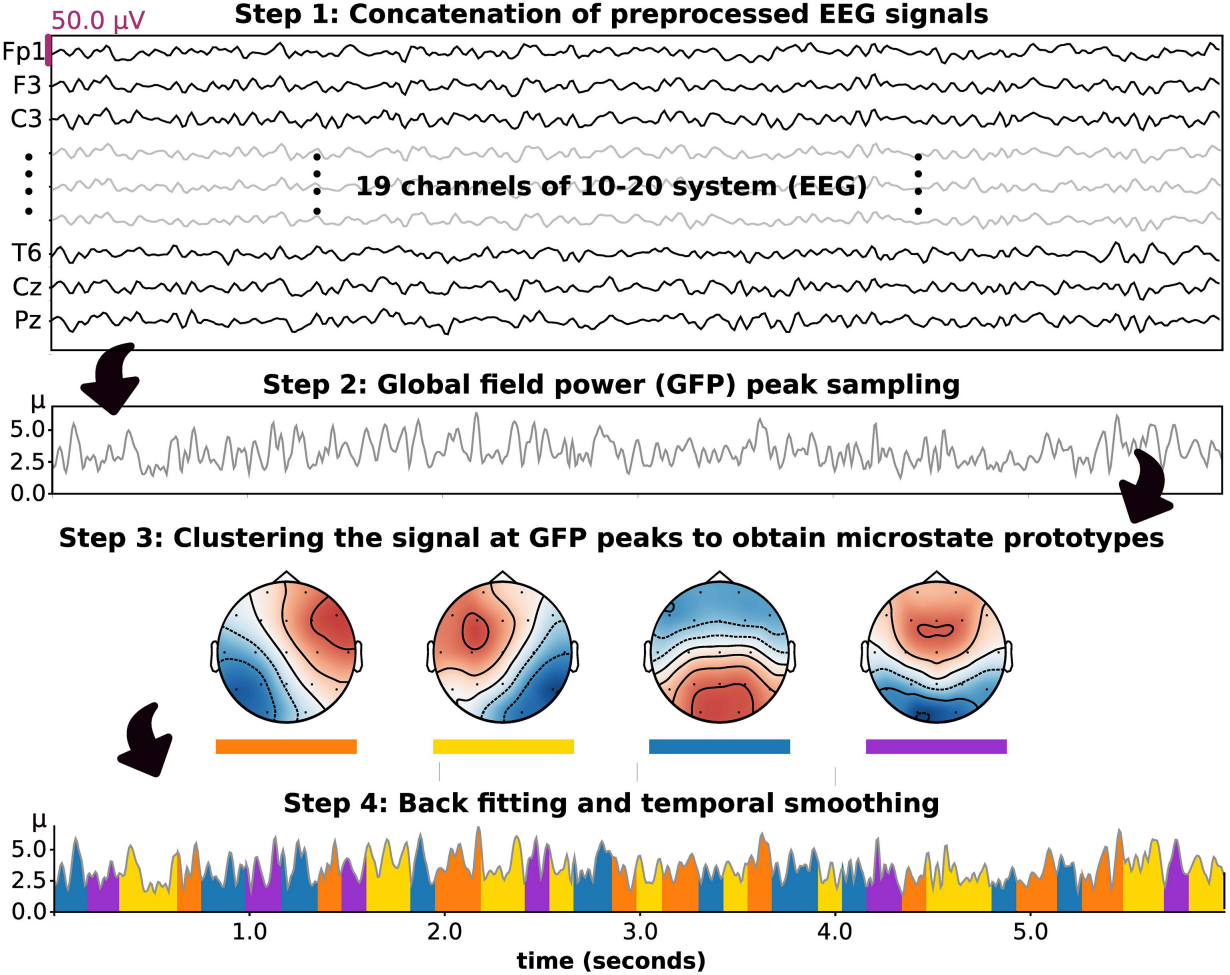
The stepwise methodology of microstate analysis introduced based on the application in this study. Four prototypes were set and included in clustering, backfitting, and temporal smoothing.

The time-domain EEG topographical map has been shown to contain some quasistable patterns whose period varies from 80 to 120 ms (Lehmann et al., 1987). These patterns have also been suggested to indicate “*the atom of thought”* underlying EEG signal activity. This idea of microstates in EEG can be represented by Equation (1),


(1) $$\begin{array}{r} {\text{s}}_{n}=i_{kn}{\text{m}}_{k}+{\text{a}}_{n},\text{ for}\;k=l_{n}\text{ and}\;n=1\ldots N. \end{array}$$

s_*n*_ represents a column vector of the EEG signal at the *n*^*th*^ sample. m_*k*_ indicates the *k*^*th*^ microstate prototype. *i*_*kn*_ is the intensity or amplitude value of the *k*^*th*^ microstate prototypes at the *n*^*th*^ sample, which must be zero except under certain circumstances, in which it equals one $(i_{kn}\times i_{k^{\prime }n}=0$, for *k* ≠ *k*′). a_*n*_ refers to a zero mean noise of the *n*^*th*^ sample. *l*_*n*_ is a class label of *n*^*th*^ EEG sample, which is determined by the most similar *k*^*th*^ prototype. *N* is a number of EEG samples. In practical computations, these microstate patterns have been investigated by using unsupervised machine learning algorithms such as modified k-means clustering. This algorithm was developed based on the traditional k-means algorithm to achieve polarity-invariant clustering and microstate-intensity modeling (Pascual-Marqui et al., 1995; Poulsen et al., 2018). In this study, we introduced the microstate analysis concept based on the application in this paper, as summarized in Figure 1.

#### 2.6.1. Microstate Prototype Computation

We decided to include four microstate prototypes in our analysis, as suggested in a prior study (Poulsen et al., 2018). To perform microstate analysis, the processed EEGs in each group were concatenated, and a rereferencing method was applied to obtain the average reference signal. The signal was then downsampled with the peak of global field power (GFP), which represents the standard deviation of **s**_*n*_. To improve the efficiency of the clustering algorithm, each EEG sample (19 channels, microvolt unit) at the peak of GFP was transformed to a vector of Z-score (19 channels) and passed to the modified k-means algorithm as an input. Hence, all microstate patterns as outputs of this model were characterized by Z-score hereafter. The concept of modified k-means algorithm is based on the minimization of the loss function (*E*) (Pascual-Marqui et al., 1995; Poulsen et al., 2018), as presented in Equation (2),


(2) $$\text{E}=\sum_{n=1}^{N}\left({\left\|{\text{s}}_{n}\right\|}^{2}-{\left({\text{s}}_{n}^{\text{T}}{\text{m}}_{k}\right)}^{2}\right).$$

In this study, we intended to reduce the computational complexity by maximizing the intensity (*i*_*kn*_) in stead of minimizing loss function (*E*). In the first iteration, the microstate prototypes were randomly initialized under these constraints: ‖m_*k*_‖ = 1 and ${\left({\text{m}}_{k}^{\text{T}}{\text{m}}_{k^{\prime }}\right)}^{2}<1$, for *k* ≠ *k*′. Then, the class label (*l*_*n*_) of the *n*^*th*^ EEG sample was acquired by maximizing the intensity (*i*_*kn*_), as shown in Equations (3) and (4):


(3) $$\begin{array}{r} i_{kn}={\text{s}}_{n}^{\text{T}}{\text{m}}_{k} \end{array}$$

(4) $$\begin{array}{r} l_{n}=\arg\underset{k}{\max}\left(i_{kn}^{2}\right). \end{array}$$

The *k*^*th*^ prototypes (m_*k*_) and class label of the *n*^*th*^ sample (*l*_*n*_) were updated in each iteration until the convergence criterion was met. In detail, The iteration was stopped when the relative change in the error of activation between iterations is less than 10^−6^. Noted that if this algorithm did not converge, it was stopped at the 1, 000^*th*^ iteration. After the criterion was met, which ended a run of the iterations, the cosine similarity (CS_*n*_) of the label prototype (m_*l*_*n*__) and the *n*^*th*^ sample (s_*n*_) was computed to evaluate their similarity. Additionally, the global explained variance (*GEV*) was calculated as the cosine similarity weighted by the fraction of the *n*^*th*^ sample's squared *GFP* and the total squared *GFP*, denoted in Equations (5) and (6):


(5) $${\text{CS}}_{n}=\frac{{\text{m}}_{k}^{\text{T}}{\text{s}}_{n}}{\left\|{\text{m}}_{k}\right\|\left\|{\text{s}}_{n}\right\|},\text{ for }k=l_{n}$$

(6) $$\text{GEV}=\sum_{n=1}^{N}\text{GE}{\text{V}}_{n}=\sum_{n=1}^{N}\left(\text{C}{\text{S}}_{n}^{2}\frac{\text{GF}{\text{P}}_{n}^{2}}{\sum_{n^{\prime }=1}^{N}\text{GF}{\text{P}}_{n^{\prime }}^{2}}\right).$$

After performing several runs with different random initializations, the run with the maximum GEV was chosen. Moreover, the most similar set of microstate prototypes for each participant group was identified.

#### 2.6.2. Microstate Parameter Calculation

After obtaining the microstate prototypes for each subject group, it was important to ensure that the prototypes were properly fitted to where they belonged. Backfitting was performed to reassign an optimal microstate label to an EEG sample based on their similarity, which can be computed using Equation (5). Because EEG noise can contribute to shortening microstate segments after clustering or backfitting, it was also important to smooth the microstate label based on the prior and/or following EEG sample. In this study, we used the small segment rejection method to perform temporal smoothing, as introduced by an earlier study (Poulsen et al., 2018). These two steps, backfitting and temporal smoothing, were applied to an individual EEG to calculate the following microstate parameters. These included the GEV, similarity (also called cosine similarity), coverage, duration, and occurrence. In detail, occurrence indicates the average frequency of microstate prototype activation (Hz). Duration is the average period of a given microstate throughout an EEG signal (milliseconds). The coverage reflects the total duration of a microstate divided by all signal periods (%). These parameters were used to perform statistical analysis hereafter. Moreover, to observe differences in microstate patterns between subject groups, we also calculated the microstate prototypes in each individual EEG signal and statistically compared the corresponding topographical maps.

### 2.7. Statistical Analysis

The SciPy statistics library in Python was applied to perform descriptive and inferential statistics (Virtanen et al., 2020). In detail, the normality of the distribution of variables was investigated using the Shapiro–Wilk test. The median and interquartile range (IQR) are used to describe non-parametric variables, while parametric variables are described with the mean and standard deviation. To perform Z-score analysis, the global mean power ratios were compared; if they were found to be significantly different, regional comparisons were performed afterward. The 19 EEG channels were reorganized into nine regions by representing each region with the mean electrode signal. These regions are the left frontal (Fp1, F3, and F7), right frontal (Fp2, F4, and F8), frontal (Fp2, F4, F8, Fz, Fp1, F3, and F7), central (C3, Cz, and C4), left temporal (T3 and T5), right temporal (T4 and T6), temporal (T4, T6, T3, and T5), parietal (P3, P4, and Pz), and occipital (O1 and O2) regions. The permutation-based *t*-test was utilized to compare microstate parameters and Z-score power ratios between independent groups. A *p*-value less than 0.05 was considered significantly different. We also used topographic analysis of variance (TANOVA) to compare microstate prototypes between groups. TANOVA is suitable for comparing topographical maps without polarity considerations because it is performed based on cosine spatial distance and permutation test (Murray et al., 2008). As the MIS is ordinal data, we used the Mann–Whitney *U*-test to compare the median between groups. The correlations among MIS, microstate parameters, and Z-score delta/theta ratio were also calculated using Spearman's rank order correlation, in which ρ (rho) >0.5 or <-0.5 was considered a strong correlation (Chan, 2003). If an EEG biomarker for MICS was suggested, logistic regression or linear regression analysis was utilized to check the accuracy in predicting MICS by using this biomarker as a feature. Train-test split validation was performed to calculate the accuracy. The receiver operating characteristic (ROC) curve was also plotted to evaluate the performance of the model.

## 3. Results

Following a recruitment period, fifty participants were enrolled in this study, but only forty-six were finally included. Three subjects (all female, two with ESRD-H and one with ESRD-L) were excluded because their EEG signals were contaminated by irreparable noise. The last subject, a male with ischemic stroke, was also excluded. Thus, twenty three subjects were allocated to each group. The demographic information including general information, clinical features, and serum chemistry result of the participants is shown in Table 1. We found that age, dialysis duration, blood urea nitrogen (BUN), creatinine, and estimated glomerular filtration rate (eGFR) were not significantly different between ESRD-H and ESRD-L patients, suggesting that their EEGs should be comparable.

**Table 1:** The demographic information of 46 subjects shows that the weight and BMI of ESRD-L patients were significantly higher than those of ESRD-H patients (^*^p ≤ 0.001), while other parameters were not different between groups, as compared by the permutation-based *t*-test.

**General information**	**ESRD-L**	**ESRD-H**				
Number of subjects	23	23				
Sex (F/M)	16 (69.6) / 7 (30.4)	15 (65.2) / 8 (34.8)				
Diabetes (yes/no)	13 (56.5) / 10 (43.5)	14 (60.9) / 9 (39.1)				
Handedness (L/R)	5 (21.7) / 18 (78.3)	5 (21.7) / 18 (78.3)				
Hypertension (yes/no)	16 (69.6) / 7 (30.4)	16 (69.6) / 7 (30.4)				
Hyperlipidemia (yes/no)	7 (30.4) / 16 (69.6)	7 (30.4) / 16 (69.6)				
Dialysis (MHD/PD)	10 (43.5) / 13 (56.5)	11 (47.8) / 12 (52.2)				
**Clinical features**	**ESRD-L**	**ESRD-H**	**t**	**p**		
Age (years)	57.57 ± 14.88	59.13 ± 11.77	0.396	0.697		
Dialysis duration (years)	6.43 ± 7.02	7.56 ± 8.53	0.487	0.635		
Weight (kg)	71.74 ± 12.4	54.77 ± 11.3	−4.854	<0.001^*^		
BMI (kg/m^2^)	26.33 ± 3.75	22.26 ± 3.45	−3.834	0.001^*^		
BUN (mg/dL)	41.51 ± 12.25	43.18 ± 18.62	0.358	0.726		
Creatinine (mg/dL)	9.01 ± 2.55	8.31 ± 2.97	−0.857	0.406		
eGFR (ml/min/1.73 m^2^)	5.76 ± 1.51	6.14 ± 2.57	0.618	0.558		
**Serum chemistry**	**ESRD-L**	**ESRD-H**	**t**	**p**	**⇓**	**⇑**
Albumin (mg/dL)	4.11 ± 0.5	3.72 ± 0.66	−2.236	0.031^*^	22 : 61	0 : 0
Calcium (mg/dL)	9.17 ± 1.09	8.94 ± 1.3	−0.655	0.516	35 : 48	17 : 17
Phosphate (mg/dL)	4.57 ± 1.18	4.23 ± 0.85	−1.103	0.276	4 : 4	48 : 52
Sodium (mEq/L)	138.02 ± 3.76	136.86 ± 4.33	−0.971	0.340	13 : 22	26 : 22
Potassium (mEq/L)	4.28 ± 0.67	4.04 ± 0.78	−1.151	0.257	9 : 26	13 : 9
Chloride (mEq/L)	96.47 ± 4.05	95.52 ± 4.3	−0.773	0.451	61 : 65	0 : 0
Bicarbonate (mEq/L)	26.2 ± 2.51	27.25 ± 2.68	1.369	0.177	4 : 0	13 : 22
Iron (μg/dL)	71.81 ± 29.65	54.82 ± 18.12	−2.345	0.012^*^	35 : 61	4 : 0
TIBC (μg/dL)	219.09 ± 29.69	200.0 ± 47.69	−1.629	0.109	83 : 74	0 : 0
Tf saturation (%)	32.92 ± 12.2	28.6 ± 11.31	−1.245	0.222	22 : 43	22 : 30
Pre-albumin (mg/dL)	34.28 ± 7.79	32.7 ± 7.43	−0.705	0.485	0 : 4	48 : 39

*Serum albumin and iron were significantly different between subject groups*

(* *p < 0.05)*.

*To describe general information, the values of dichotomous variables are presented with their percentages in parentheses (%). The serum components are interpreted as lower (⇓) and higher (⇑) than normal range values and is reported as the percentage of ESRD-L subjects to the percentage of ESRD-H subjects (%L:%H)*.

### 3.1. Participant MICS Status

After evaluating the MICS status of the participants with the MIS, we found that the causes of significant differences between the ESRD-H and ESRD-L groups included the quantity of dietary intake, gastrointestinal symptoms, loss of subcutaneous fat, muscle wasting, serum albumin, and TIBC. As seen in Table 2, the second and third components of the MIS show that more than 26% of the high-risk group had slightly decreased appetite and occasionally felt nauseated. The sixth and seventh components indicated that more than 75% of ESRD-H patients had mild to moderate muscle wasting and subcutaneous fat loss. The ninth and tenth components signified that more than 45% of ESRD-H patients had low serum albumin (less than 3.4 g/dL) and TIBC levels (less than 200 μg/dL). The outcome of the blood test also confirmed the significant difference in serum albumin and iron (*p* < 0.04), but TIBC was not significantly different due to the high deviation in values. The remaining electrolytes were also not significantly different between groups. Interestingly, greater proportions of ESRD-H patients had hypoalbuminemia and hypoironemia than ESRD-L patients (Table 1). The levels of serum albumin (ρ = −0.628, *p* < 0.001) and iron (ρ = −0.317, *p* < 0.032), BMI (ρ = −0.679, *p* < 0.001) and weight (ρ = −0.717, *p* < 0.001) were also found to be negatively correlated with the MIS.

**Table 2:** The MIS for questions 2, 3, 6, 7, 8, and 9 was significantly different between groups [median (IQR), ^*^p < 0.05].

**Question**	**Score frequency**								**MIS**		***U*1**	**p**
	**ESRD-L (%)**				**ESRD-H (%)**				**Low (** ≤ 5**)**	**High (>5)**		
	**0**	**1**	**2**	**3**	**0**	**1**	**2**	**3**				
1									0 (0.0, 0.0)	0 (0.0, 0.0)	290.5	0.395
2	100^**^				70^**^	30			0 (0.0, 0.0)	0 (0.0, 1.0)	345	0.005^*^
3	100^**^				74^**^	26			0 (0.0, 0.0)	0 (0.0, 0.5)	333.5	0.010^*^
4									0 (0.0, 0.0)	0 (0.0, 1.0)	322	0.068
5									1 (1.0, 2.0)	1 (1.0, 2.0)	251.5	0.760
6	74^**^	26			17	61^**^	22		0 (0.0, 0.5)	1 (1.0, 1.0)	429	<0.001^*^
7	74^**^	26			22	52^**^	26		0 (0.0, 0.5)	1 (1.0, 1.5)	420.5	<0.001^*^
8									0 (0.0, 0.0)	0 (0.0, 0.0)	310	0.091
9	61^**^	26	13		39	13	43^**^	4	0 (0.0, 1.0)	1 (0.0, 2.0)	352	0.038^*^
10	17	70^**^	9	4	17	13	57^**^	13	1 (1.0, 1.0)	2 (1.0, 2.0)	384.5	0.005^*^
total									4 (3.0, 4.5)	7 (6.0, 9.0)	529	<0.001^*^

*These questions reflect the following respective factors: quantity of dietary intake; gastrointestinal symptoms; loss of subcutaneous fat; muscle wasting; serum albumin; and TIBC. The score frequencies are shown for the statistically significant questions in percentages, with the highest values labeled by double asterisks*

(**). *The p-value and U1 were calculated by the Mann–Whitney U-test*.

### 3.2. Delta/Theta Ratio in ESRD With MICS

**Figure 2:**
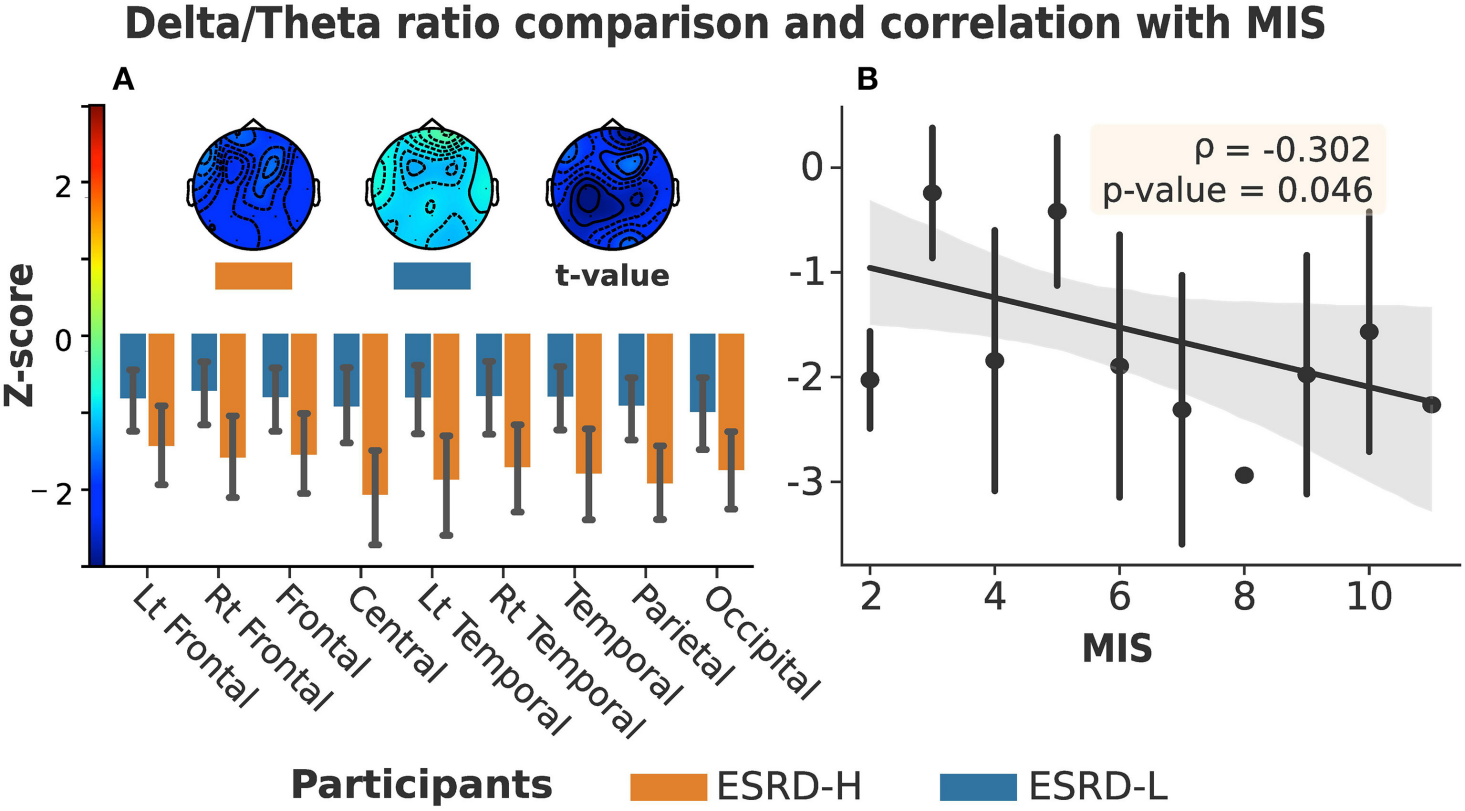
**(A)** Delta/theta ratio in all regions and channels exhibited significant differences between ESRD-H (orange) and ESRD-L (blue). (The box's rim: Z-score mean, whiskers: 95% CI, Topographical maps: Z-score mean and *t*-value of pairwise comparison). **(B)** No significant correlation was found between MIS and delta/theta ratio (Gray: 95% CI, point: Z-score mean, vertical line: SD).

The global delta/theta ratio of the ESRD-H group was lower than that of the ESRD-L group (*t* = −2.613, *p* = 0.011). We found that all electrode regions exhibited differences between the ESRD-H and ESRD-L groups (−2.660 ≤ *t* ≤ −2.151, 0.011 ≤ *p* ≤ 0.037). Pairwise comparison subsequently confirmed differences for each electrode (−3.082 ≤ *t* ≤ −2.092, 0.004 ≤ *p* ≤ 0.041). Since a Z-score of zero refers to a healthy control (HC), we compared the Z-score to zero and found a strong, significant deviation in the delta/theta ratio throughout both groups as demonstrated in Figure 2A (ESRD-L: −4.795 ≤ *t* ≤ −2.753, *p* < 0.009, ESRD-H: −7.247 ≤ *t* ≤ −3.264, *p* < 0.002). An insignificant correlation between the delta/theta ratio and the MIS was also observed (ρ = −0.302, *p* = 0.046) as presented in Figure 2B.

### 3.3. Microstate Findings in ESRD With MICS

#### 3.3.1. Microstate Prototype Findings

**Figure 3:**
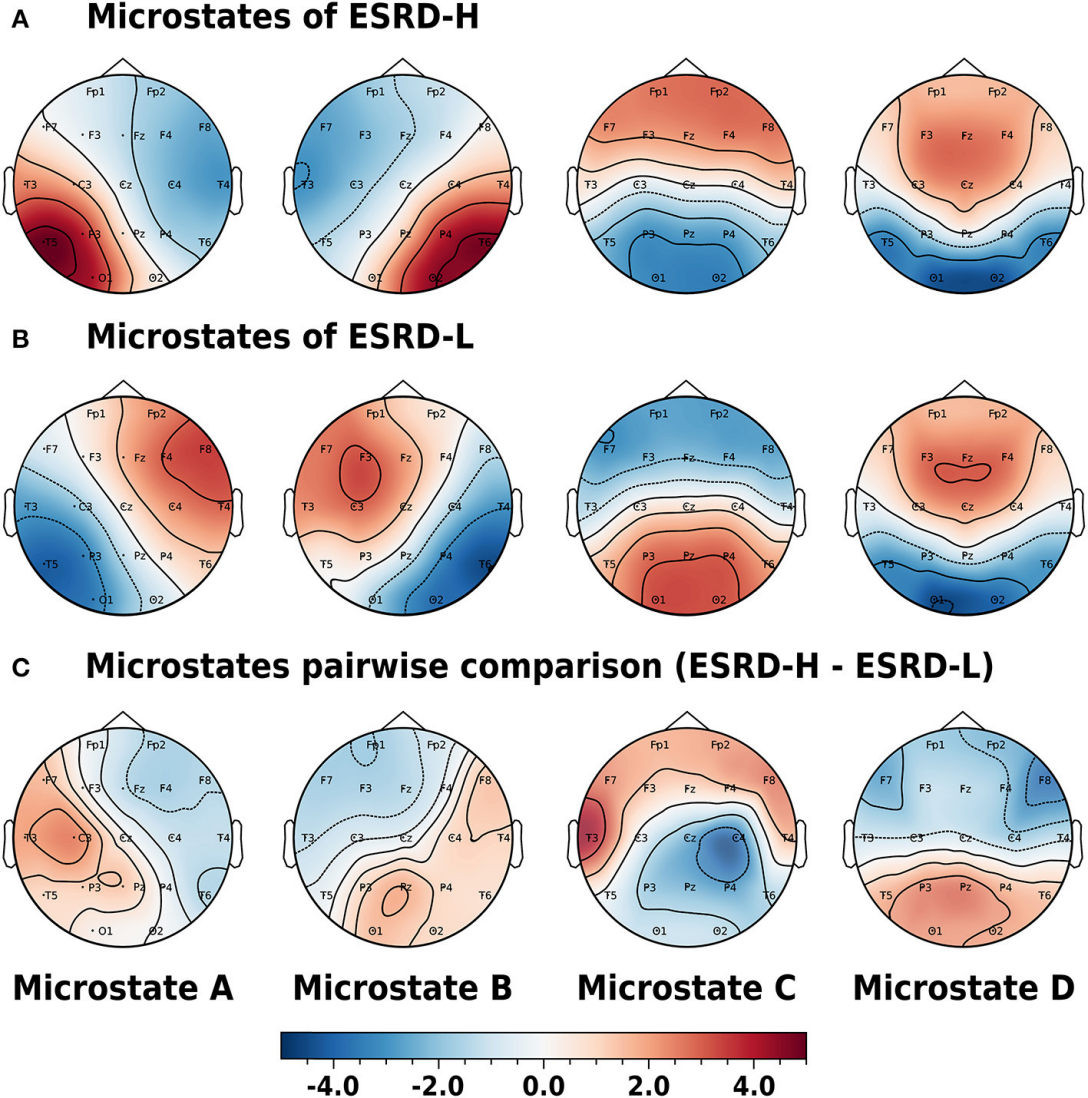
Phase-independent microstate prototypes characterized by Z-score are orderly demonstrated in the A, B, C, and D patterns of the **(A)** ESRD-H and **(B)** ESRD-L groups. **(C)** Pairwise comparisons for each channel are represented by *t*-values (ESRD-H - ESRD-L). No statistically significant differences were observed in any of the topographic maps. All values are represented by color bars.

The microstate prototypes we archived from each subject group are shown in Figures 3A,B. These prototypes can globally explain 77.47±5.98 and 74.23±5.57% of the selected EEGs of the ESRD-H and ESRD-L groups, respectively (*t* = −1.551, *p* = 0.124). The average similarities were 65.48±4.23 and 62.34±2.44%, respectively (*t* = −1.012, *p* = 0.318). Using TANOVA, we found that these patterns were not significantly different between groups; the *p*-values for prototypes A to D were 0.120, 0.852, 0.260, and 0.480, and the *t*-values from the pairwise comparisons are shown in Figure 3C. Note that we had already adjusted the phase of each sample before comparing them.

#### 3.3.2. Microstate Parameter Findings

**Figure 4:**
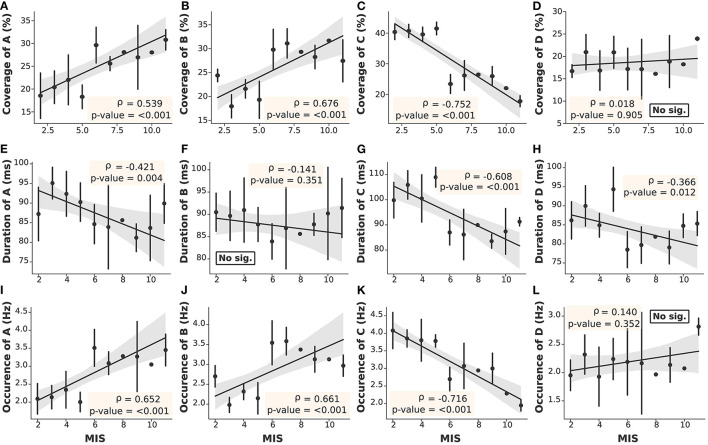
The correlation between the MIS and microstate parameters in each prototype was obtained by Spearman's rank order correlation test. With the exceptions of **(D,F,L)**, the remaining parameters **(A–C,E,G,H–K)** demonstrated significant correlations with the MIS (Gray: 95% CI, point: Z-score mean, vertical line: SD).

The microstate parameters analyzed in this study were coverage, duration, and occurrence, as shown in Table 3. We found that all parameters in ESRD-L were noticeably imbalanced, as microstate C was the most prominent pattern, overwhelming the remaining classes; in contrast, microstate D was the least dominant class in ESRD-H. These findings inspired us to compare parameters between the ESRD-H and ESRD-L groups. We found that coverage, duration, and occurrence were significantly different for most microstate prototypes (*p* ≤ 0.001); however, microstate D coverage and occurrence and microstate B duration were not different between groups. To further analyze the relationship between the MIS and the parameters of each microstate pattern, we calculated their correlations and trends. Notably, microstate D coverage and occurrence and microstate B duration not only were not significantly different between groups but also were not significantly correlated with the MIS. Fair to strong correlations were found for the remaining parameters, as shown in Figure 4 (Negative: −0.75 ≤ ρ ≤ −0.37, Positive: 0.54 ≤ ρ ≤ 0.68, *p* < 0.012).

**Table 3:** Significant differences were observed for most prototypes and parameters.

**Parameters**	**Class**	**ESRD-H**	**ESRD-L**	***t*** or ***U*1**	**p**
Coverage (%)	A	28.17 ± 4.57	19.55 ± 5.62	5.58	<0.001^*^
	B	29.63 ± 3.58	19.77 ± 4.59	7.95	<0.001^*^
	C	23.53 ± 4.42	40.76 ± 2.80	−15.44	<0.001^*^
	D	18.67 ± 6.17	19.92 ± 6.52	−0.65	0.525
Duration (ms)	A	84.99 ± 6.84	92.19 ± 5.96	−3.72	0.001^*^
	B	87.09 ± 6.78	90.48 ± 6.81	−1.66	0.107
	C	87.46 ± 6.67	106.13 ± 11.97	−6.39	<0.001^*^
	D	81.65 ± 7.48	89.79 ± 6.96	−3.74	0.001^*^
Occurrence (Hz)	A	3.0 (3.0, 4.0)	2.0 (2.0, 3.0)	469	<0.001^*^
	B	3.0 (3.0, 4.0)	2.0 (2.0, 2.0)	488.5	<0.001^*^
	C	3.0 (2.0, 3.0)	4.0 (4.0, 4.0)	49.5	<0.001^*^
	D	2.0 (2.0, 3.0)	2.0 (2.0, 2.0)	279	0.701

*Because occurrence is a discrete parameter, its median was compared by the Mann–Whitney U-test; the permutation-based t-test was used to compare means between the remaining parameters*

(* *p < 0.05)*.

#### 3.3.3. Correlation Between Delta/Theta Ratio and Microstate Parameters

**Figure 5:**
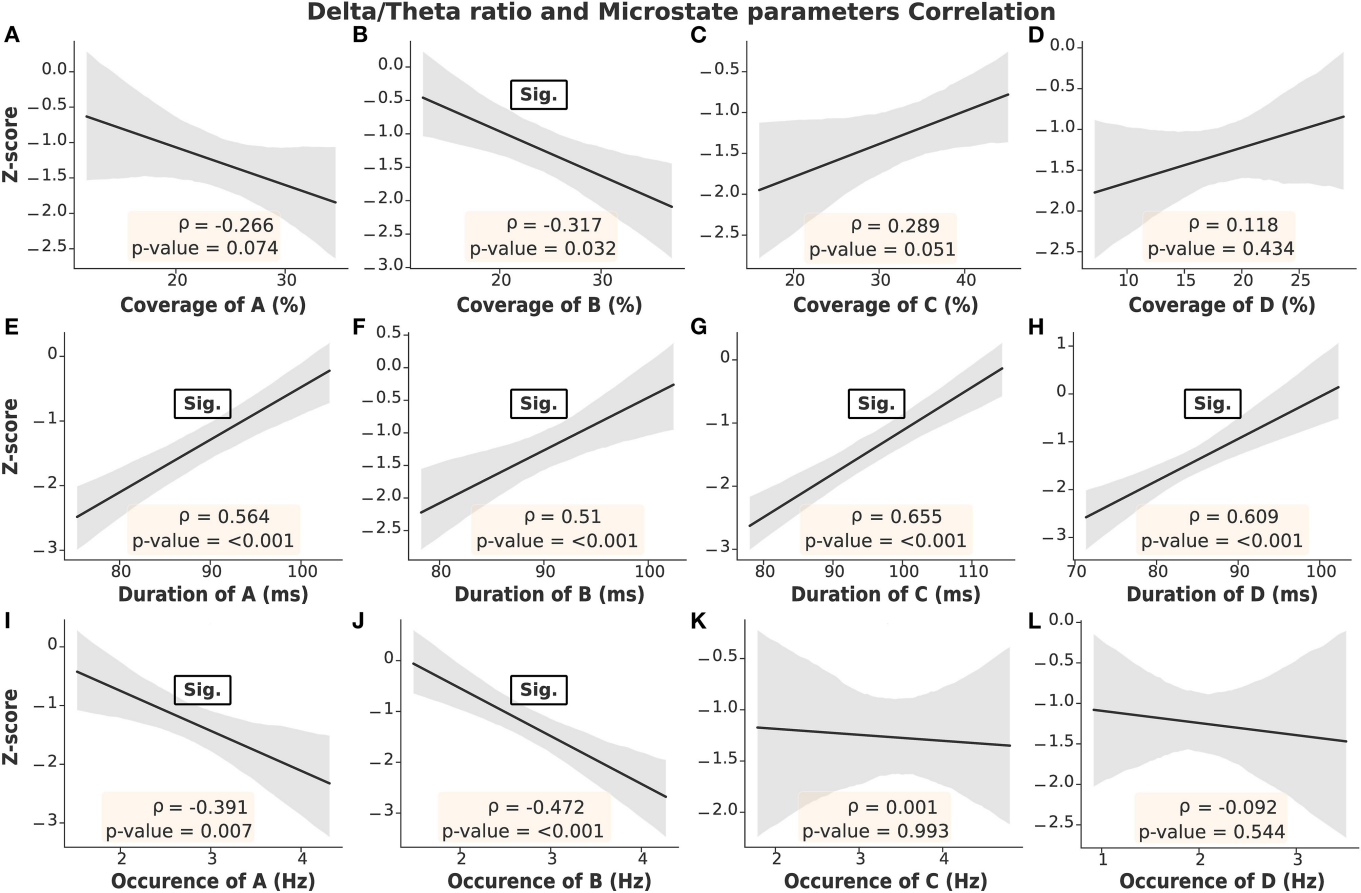
Strong correlations were obtained between the delta/theta ratio and the duration of each microstate, as seen in **(E–H)**. Fair correlations are shown in **(B,I,J)**. On the other hand, insignificant correlations were found in **(A,C,D)**. Gray: 95% CI.

As mentioned above, the delta/theta ratio was not significantly correlated with the MIS; however, it was significantly different between the ESRD-H and ESRD-L groups. Thus, we chose to further investigate the correlations between the microstate parameters and delta/theta ratio to find some reasonable explanations for this significant difference. As seen in Figure 5, we found that the delta/theta ratio was strongly positively correlated with the duration of each microstate (0.51 ≤ ρ ≤ 0.66, *p* < 0.001) and fairly negatively correlated with the occurrences of A and B and the coverage of B (−0.47 ≤ ρ ≤ −0.32, *p* < 0.04). These findings led us to further calculate the correlation between the average duration of all microstates and the delta/theta ratio.

### 3.4. Suggested Biomarkers for MICS and Encephalopathy

**Figure 6:**
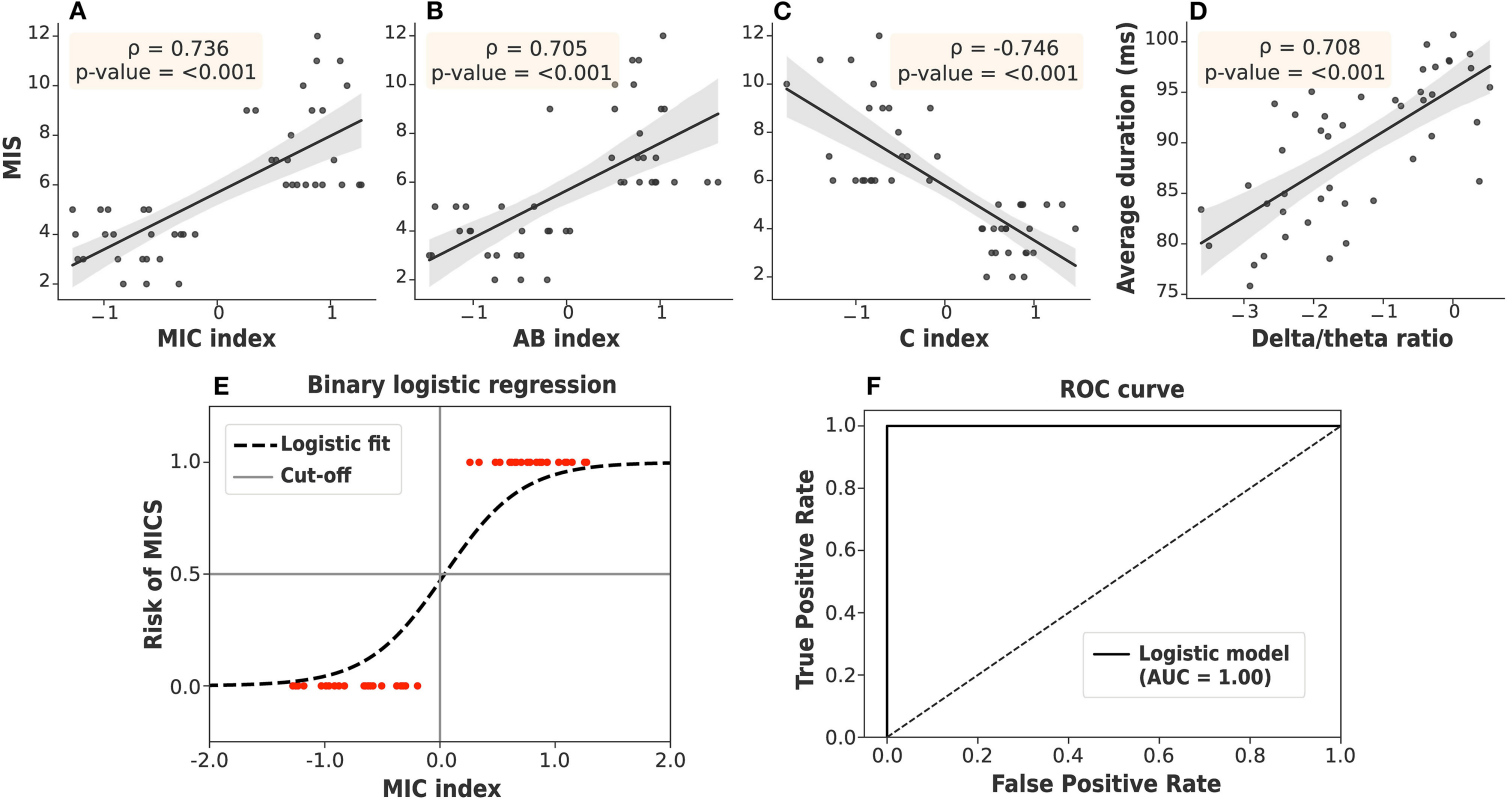
**(A)** The MIC index, **(B)** AB index, and **(C)** C index were strongly correlated with the MIS. **(D)** The delta/theta ratio was also strongly correlated with the average duration. **(E)** The MIC index, as a feature in binary logistic regression, discriminated ESRD patients at high and low risk of MICS with 100% accuracy by using train-test split validation. **(F)** The ROC curve demonstrated a 100% area under the curve (AUC). Gray: 95% CI.

After determining the correlation between the MIS and the microstate parameters, we noticed positive correlations for the coverage and occurrence of microstates A and B (0.54 ≤ ρ ≤ 0.68, *p* < 0.001) and stronger negative correlations for the coverage, occurrence, and duration of microstate C (−0.75 ≤ ρ ≤ −0.61, *p* < 0.001), as seen in Figure 4. These findings motivated us to find an optimal index that could represent all malnutrition-related features of the microstates. Consequently, we proposed the “Malnutrition-Inflammation Cutoff Index” (MIC index), which combines the significant microstate A and B parameters (AB index) and C parameters (C index), as shown in Equations (7)–(9):


(7) $$\begin{array}{r} {\text{AB}}_{i}=\frac{Z\left({\text{Occ}}_{i}^{\text{A}}+{\text{Occ}}_{i}^{\text{B}}\right)+Z\left({\text{Cov}}_{i}^{\text{A}}+{\text{Cov}}_{i}^{\text{B}}\right)}{2} \end{array}$$

(8) $$\begin{array}{r} {\text{C}}_{i}=\frac{Z\left({\text{Occ}}_{i}^{\text{C}}\right)+Z\left({\text{Cov}}_{i}^{\text{C}}\right)+Z\left({\text{Dur}}_{i}^{\text{C}}\right)}{3} \end{array}$$

(9) $$\begin{array}{r} {\text{MIC}}_{i}=\frac{{\text{AB}}_{i}-{\text{C}}_{i}}{2}, \end{array}$$

where ${\text{Occ}}_{i}^{k}$, ${\text{Cov}}_{i}^{k}$, and ${\text{Dur}}_{i}^{k}$ represent the occurrence, coverage, and duration of microstate *k* (*A*, *B*, or *C*) in the *i*^*th*^ subject. *Z* indicates the transformation to the Z-score, which relies on the mean and SD of the population. As seen in Figures 6A–C, both the C and AB indices were significantly correlated with the MIS. However, the AB index alone cannot clearly separate the ESRD-H and ESRD-L groups. The C index can distinguish between the groups effectively, but it may not be specific for malnutrition-inflammation because a reduction in microstate C duration has also been observed in other diseases, such as head injury, dementia, and Alzheimer's disease (Nishida et al., 2013; Khanna et al., 2015; Michel and Koenig, 2018). Thus, we combined the AB and C indices into the MIC index. We noticed that a positive MIC index possibly reflected a high risk of MICS in ESRD patients. To confirm this idea, we used the MIC index as a feature in binary logistic regression and validated it by using the train-test split method. Based on the data from our 46 subjects, zero cutoff of this EEG biomarker discriminated ESRD-H (MIS > 5) and ESRD-L (MIS ≤ 5) with 100% accuracy, sensitivity, and specificity, as shown in Figures 6E,F.

The findings on the correlation between the delta/theta ratio and duration of each microstate were likewise of interest (Figure 5). Upon further investigation, we found that the average duration of all microstates was positively correlated with the delta/theta ratio (ρ = 0.708, *p* < 0.001), as shown in Figure 6D. Thus, the average microstate duration may correspondingly be considered an alternative biomarker for encephalopathy.

## 4. Discussion

### 4.1. Main Findings Summary and a Conflict With the Hypothesis

In this study, we aimed to investigate both the delta/theta ratio and EEG microstates in ESRD-H and ESRD-L patients. Moreover, we attempted to determine the correlations among the MIS, delta/theta ratio, and microstate parameters to potentially clarify their relationships. After thorough investigation and analysis, we summarize our findings as follows. Subcutaneous fat loss, muscle wasting, less appetite, and slight gastrointestinal symptoms were observed in ESRD-H (fairly) and ESRD-L (slightly). These complications may contribute to the lower BMI and weight in ESRD-H than in ESRD-L. In addition, iron and albumin deficiency was found to be associated with a high risk of MICS, as evaluated by MIS and blood chemistry tests. The delta/theta ratio was lowest in ESRD-H, followed by ESRD-L and healthy controls, but it may not be significantly correlated with the MIS. However, this ratio was strongly correlated with the individual and average microstate duration. Thus, we suggest that the average duration of all microstates may be another encephalopathic biomarker candidate. Furthermore, we found that the patterns of the microstate prototypes were not significantly different between ESRD-H and ESRD-L. Most microstate parameters, however, were different between groups and were also correlated with the MIS, including the coverage and occurrence of A and B (strong negative) and all C parameters (strong positive). By combining these significant parameters, we developed and proposed the MIC index as an optimal biomarker for distinguishing ESRD patients at high or low risk of MICS. This biomarker was evaluated for its classification efficiency by using it as a feature in a binary logistic regression model. This classification on our data then yielded 100% accuracy by train-test split validation and 100% sensitivity and specificity from the ROC curve.

After considering all our findings, it appears that in one aspect, our hypothesis may not correct obviously. As hypothesized, the delta/theta ratio may be correlated with the MIS; however, we observed a fair correlation (ρ = −0.302), but it was nearly insignificant (*p* = 0.046). Although MICS can induce encephalopathy (Kalantar-Zadeh et al., 2003; Hamed, 2019), this cerebral complication is a multifactorial disorder; hypertension, diabetes, uremic toxin accumulation, and metabolic deterioration are other potential causes (Britton et al., 2016; Hamed, 2019; Jatupornpoonsub et al., 2021). Therefore, we suggest that the delta/theta ratio could probably assess encephalopathy in ESRD patients with high and low risk of MICS (as we found a significant difference between ESRD-H and ESRD-L), but this ratio cannot indicate the severity of MICS specifically.

### 4.2. Why Could the MIC Index Distinguish the Risk of MICS in ESRD Patients?

We suggest that microstates A, B, and C, which refer to the phonological, visual, and salience networks, respectively (Mantini et al., 2007; Britz et al., 2010; Nishida et al., 2013), may be the causes of the significant associations observed in this study, resulting in the formation of the MIC index. In detail, low BMI, iron deficiency, and hypoalbuminemia have been suggested to be associated with cognitive impairment and dementia in aging adults (Llewellyn et al., 2010; Garcia-Ptacek et al., 2014; Jáuregui-Lobera, 2014), and MICS itself was proposed to be a cause of cognitive decline (Zheng et al., 2017). A correlation between cognitive impairment and MIS was also proposed (Guenzani et al., 2019). These studies jointly implicate cognitive impairment, which is a cerebral complication involving neurodegenerative processes. As the severity of cognitive impairment increases, it may develop into Alzheimer's disease, dementia, or frontotemporal dementia (Nishida et al., 2013). An earlier study found that abnormal functioning of the salience network, which is located in the anterior insular and dorsal anterior cingulate cortices, may lead to cognitive impairment (Menon, 2020); in addition, the phonological network, which is located in the temporal region, has also exhibited alterations in cognitive impairment and Alzheimer's disease (Nishida et al., 2013; Musaeus et al., 2019, 2020). Therefore, microstates A and C may be reasonably associated with MICS.

Cognitive impairment, lower appetite, and mild depression were found to be symptoms of early encephalopathy, which can be induced by MICS (Kalantar-Zadeh et al., 2003; Hamed, 2019). Although encephalopathy has a multifactorial pathogenesis, the delta/theta ratio tends to decrease in severe encephalopathy of any pathogenic mechanism (Faigle et al., 2013; Kamiya-Matsuoka and Tummala, 2017; Jatupornpoonsub et al., 2021). In this study, the correlation between the delta/theta ratio and the average duration of all microstates indicated that the average microstate duration may be reduced in more severe encephalopathy. Another study also found that a reduction in the average microstate duration was associated with cognitive decline and depression, which these symptoms also related to encephalopathy (Khanna et al., 2015). Hence, the average microstate duration may be considered another alternative encephalopathic biomarker. The delta/theta ratio is not only positively correlated with the average duration but also negatively correlated with the coverage (ρ = −0.32, *p* < 0.032) and occurrence (ρ = −0.47, *p* < 0.001) of microstate B. This may indicate that these parameters may be higher in severe encephalopathy. In addition, encephalopathy can induce visual disturbance in ESRD patients, a condition called posterior reversible encephalopathy syndrome (PRES) that has previously been observed by MRI; studies have shown that the typical origin of abnormalities was the parieto-occipital region of the brain, which is near the visual network (Kamiya-Matsuoka and Tummala, 2017; Hamed, 2019). As a result, the alteration in the coverage and occurrence of microstate B in this study may have been caused by the visual disturbance in this encephalopathy. Thus, microstate B may also be reasonably associated with the MICS. In summary, we suggest that the MIC index could distinguish the risk of MICS in ESRD patients, as this index combines features related to encephalopathy and cognitive impairment, which can be induced by MICS.

### 4.3. Limitations and Future Works

The following limitations of our work should be considered. First, the Z-score delta/theta ratio was calculated with NeuroGuide software (commercial software), which limits our ability to provide the standard error for normal controls and reduces the repeatability of our study. The suggestion on the average duration of microstates as a biomarker needs to be carefully considered because we solely relied on the delta/theta ratio, which is an alternative method of encephalopathy assessment (not from clinical investigation). Although the delta/theta ratio can be used to evaluate the severity of encephalopathy as mentioned in a prior study, the proper cutoff value for this ratio in terms of clinical significance remains unknown. Hence, we also cannot suggest the cutoff value for the average microstate duration, which may be considered another limitation and should be addressed in future studies.

The proposed MIC index also had some limitations. Indeed, this index can only distinguish ESRD patients at high and low risk of MICS (binary classification), but it cannot imply or predict the severity of MICS as it is not a regression parameter. To calculate the value of this biomarker, the mean and standard deviation of the ESRD patient population need to be calculated at the beginning because it relies on the transformation of the microstate parameters to Z-scores (Equations 7–9). This limitation will lead to the database production of microstate parameters in the future. Although our result indicated that MIC index could accurately distinguish ESRD subjects with different risk of MICS, the robustness of this index is still questionable due to the small sample size of our study. Therefore, we also suggested to observe MIC index in the larger sample size. Another consideration regarding the relationship between microstate B parameters and encephalopathy is that we were unable to determine the specific causes of encephalopathy that altered these parameters in the participants. However, we attempted to explain our findings by using inferences from prior studies and the delta/theta ratio itself. Therefore, our recommended biomarkers need to be studied further in clinical practice.

Another limitation is that we cannot provide menstrual cycle data of recruited female subjects in this study, which lead to the consideration in two aspects. Although the effect of menstrual disturbance on appetite and iron metabolism of ESRD patients remain inconclusive in the prior study, periovulatory phase of menstrual cycle can contribute to lower quantity of food intake in female healthy adult (Strahler et al., 2020). Consequently, it may affect the second question of MIS and deviate the score to be either zero or one in our ESRD subjects (normal or slightly less quantity of food intake). Iron deficiency presented in this study might also be affected by menstruation (Peinado et al., 2021). As a result, we suggested that our results should be interpreted with these considerations. We also recommended to clarify these effects on ESRD patients in the future study.

## 5. Conclusion

Based on the delta/theta ratio findings in our study, ESRD participants at high risk of MICS presented with more severe encephalopathy. Most microstate parameters were also significantly different between groups. These differences in the delta/theta ratio and microstate parameters might be caused by the induction of MICS resulting in cognitive impairment and encephalopathy. A strong correlation between the delta/theta ratio and the average duration of microstates was observed; hence, we suggest that the average microstate duration may be a candidate encephalopathic biomarker. The strong correlation between certain microstate parameters and the MIS also inspired the MIC index as an optimal EEG biomarker for MICS. This biomarker could accurately distinguish ESRD patients at high and low risk of MICS in ESRD. Because the MIC index and average microstate duration relied solely on microstate parameters and is unlikely to require clinician intervention, we expect that these biomarkers might potentially contribute to improve ESRD patient monitoring in the future.

## Data Availability Statement

The raw data supporting the conclusions of this article will be made available by the authors, without undue reservation.

## Ethics Statement

The studies involving human participants were reviewed and approved by Institutional Review Board of Phramongkutklao Hospital with certificate of approval (COA) number S072h/62 and Institutional Review Board of Mahidol University with COA number MU-CIRB 2020/393.2511. The patients/participants provided their written informed consent to participate in this study.

## Author Contributions

TJ, PT, OS, and YW contributed to the conception and design of the study. TJ performed the experiment and statistical analysis. TJ and YW wrote the first draft of the manuscript. All authors contributed to manuscript revision and read and approved the submitted version.

## Funding

This work was funded by the National Innovation Agency (Public Organization) and Thai Union Group PCL supported this work [grant number PE0105-02-62-07-0143: Tuna coproduct nutritional supplement for chronic kidney disease patients].

## Conflict of Interest

The authors declare that this study received funding from Thai Union Group PCL. The funder was not involved in the study design, collection, analysis, interpretation of data, the writing of this article or the decision to submit it for publication.

## Publisher's Note

All claims expressed in this article are solely those of the authors and do not necessarily represent those of their affiliated organizations, or those of the publisher, the editors and the reviewers. Any product that may be evaluated in this article, or claim that may be made by its manufacturer, is not guaranteed or endorsed by the publisher.

## References

[B1] Ablin P. Cardoso J.-F. Gramfort A. (2018). Faster independent component analysis by preconditioning with Hessian approximations. IEEE Trans. Signal Process. 66, 4040–4049. 10.1109/TSP.2018.284420327295638

[B2] ANI (2018). NeuroGuide Help Manual. Largo, FL: Applied Neuroscience, Inc.

[B3] Bramania P. K. Ruggajo P. Bramania R. Mahmoud M. Furia F. F. (2020). Prevalence of malnutrition inflammation complex syndrome among patients on maintenance haemodialysis at muhimbili national hospital in tanzania: a cross-sectional study. BMC Nephrol. 21:521. 10.1186/s12882-020-02171-3 33256618 PMC7708158

[B4] Britton J. W. Frey L. C. Hopp J. L. Korb P. Koubeissi M. Z. Lievens W. E.. (2016). Electroencephalography (EEG): An Introductory Text and Atlas of Normal and Abnormal Findings in Adults, Children, and Infants. American Epilepsy Society, Chicago, IL. 27748095

[B5] Britz J. Van De Ville D. Michel C. M. (2010). BOLD correlates of EEG topography reveal rapid resting-state network dynamics. Neuroimage 52, 1162–1170. 10.1016/j.neuroimage.2010.02.052 20188188

[B6] Chan Y. (2003). Biostatistics 104: Correlational analysis. Singapore Med. J. 44, 614–619. 14770254

[B7] Faigle R. Sutter R. Kaplan P. W. (2013). The electroencephalography of encephalopathy in patients with endocrine and metabolic disorders. J. Clin. Neurophysiol. 30, 505–516. 10.1097/WNP.0b013e3182a73db9 24084183 PMC3826953

[B8] Garcia-Ptacek S. Faxén-Irving G. Čermáková P. Eriksdotter M. Religa D. (2014). Body mass index in dementia. Eur. J. Clin. Nutr. 68, 1204–1209. 10.1038/ejcn.2014.199 25271014

[B9] Gramfort A. Luessi M. Larson E. Engemann D. A. Strohmeier D. Brodbeck C.. (2013). MEG and EEG data analysis with MNE-Python. Front. Neurosci. 7:267. 10.3389/fnins.2013.00267 24431986 PMC3872725

[B10] Guenzani D. Buoli M. Caldiroli L. Carnevali G. S. Serati M. Vezza C.. (2019). Malnutrition and inflammation are associated with severity of depressive and cognitive symptoms of old patients affected by chronic kidney disease. J. Psychosom. Res. 124:109783. 10.1016/j.jpsychores.2019.109783 31443824

[B11] Hamed S. A. (2019). Neurologic conditions and disorders of uremic syndrome of chronic kidney disease: presentations, causes, and treatment strategies. Expert Rev. Clin. Pharmacol. 12, 61–90. 10.1080/17512433.2019.1555468 30501441

[B12] Harvinder G. S. Swee W. C. Karupaiah T. Sahathevan S. Chinna K. Ahmad G.. (2016). Dialysis Malnutrition and Malnutrition Inflammation Scores: screening tools for prediction of dialysis-related protein-energy wasting in Malaysia. Asia Pac. J. Clin. Nutr. 25, 26–33. 10.6133/apjcn.2016.25.1.01 26965758

[B13] Ho L. Wang H. H. Peng Y. S. Chiang C. K. Huang J. W. Hung K. Y.. (2008). Clinical utility of malnutrition-inflammation score in maintenance hemodialysis patients: focus on identifying the best cut-off point. Am. J. Nephrol. 28, 840–846. 10.1159/000137684 18535370

[B14] Jatupornpoonsub T. Thimachai P. Supasyndh O. Wongsawat Y. (2021). Background activity findings in end-stage renal disease with and without comorbid diabetes: an electroencephalogram study. Front. Hum. Neurosci. 15:579. 10.3389/fnhum.2021.741446 34690724 PMC8531714

[B15] Jáuregui-Lobera I. (2014). Iron deficiency and cognitive functions. Neuropsychiatr. Dis. Treat. 10:2087. 10.2147/NDT.S72491 25419131 PMC4235202

[B16] Kalantar-Zadeh K. Ikizler T. A. Block G. Avram M. M. Kopple J. D. (2003). Malnutrition-inflammation complex syndrome in dialysis patients: causes and consequences. Am. J. Kidney Dis. 42, 864–881. 10.1016/j.ajkd.2003.07.016 14582032

[B17] Kalantar-Zadeh K. Kopple J. D. Block G. Humphreys M. H. (2001). A Malnutrition-Inflammation Score is correlated with morbidity and mortality in maintenance hemodialysis patients. Am. J. Kidney Dis. 38, 1251–1263. 10.1053/ajkd.2001.29222 11728958

[B18] Kamiya-Matsuoka C. Tummala S. (2017). Electrographic patterns in patients with posterior reversible encephalopathy syndrome and seizures. J. Neurol. Sci. 375, 294–298. 10.1016/j.jns.2017.02.017 28320152

[B19] Khanna A. Pascual-Leone A. Farzan F. (2014). Reliability of resting-state microstate features in electroencephalography. PLoS ONE 9:e114163. 10.1371/journal.pone.0114163 25479614 PMC4257589

[B20] Khanna A. Pascual-Leone A. Michel C. M. Farzan F. (2015). Microstates in resting-state EEG: current status and future directions. Neurosci. Biobehav. Rev. 49, 105–113. 10.1016/j.neubiorev.2014.12.010 25526823 PMC4305485

[B21] Lehmann D. Ozaki H. Pál I. (1987). EEG alpha map series: brain micro-states by space-oriented adaptive segmentation. Electroencephalogr. Clin. Neurophysiol. 67, 271–288. 10.1016/0013-4694(87)90025-3 2441961

[B22] Llewellyn D. J. Langa K. M. Friedland R. P. Lang I. A. (2010). Serum albumin concentration and cognitive impairment. Curr. Alzheimer Res. 7, 91–96. 10.2174/156720510790274392 20205675 PMC2886725

[B23] Mantini D. Perrucci M. G. Del Gratta C. Romani G. L. Corbetta M. (2007). Electrophysiological signatures of resting state networks in the human brain. Proc. Natl. Acad. Sci. U.S.A. 104, 13170–13175. 10.1073/pnas.0700668104 17670949 PMC1941820

[B24] Menon V. (2020). Brain networks and cognitive impairment in psychiatric disorders. World Psychiatry 19:309. 10.1002/wps.20799 32931097 PMC7491636

[B25] Michel C. M. Koenig T. (2018). EEG microstates as a tool for studying the temporal dynamics of whole-brain neuronal networks: a review. Neuroimage 180, 577–593. 10.1016/j.neuroimage.2017.11.062 29196270

[B26] Murray M. M. Brunet D. Michel C. M. (2008). Topographic ERP analyses: a step-by-step tutorial review. Brain Topogr. 20, 249–264. 10.1007/s10548-008-0054-5 18347966

[B27] Musaeus C. S. Engedal K. Høgh P. Jelic V. Khanna A. R. Kjær T. W.. (2020). Changes in the left temporal microstate are a sign of cognitive decline in patients with Alzheimer's disease. Brain Behav. 10:e01630. 10.1002/brb3.1630 32338460 PMC7303403

[B28] Musaeus C. S. Nielsen M. S. Høgh P. (2019). Microstates as disease and progression markers in patients with mild cognitive impairment. Front. Neurosci. 13:563. 10.3389/fnins.2019.00563 31263397 PMC6584800

[B29] Nishida K. Morishima Y. Yoshimura M. Isotani T. Irisawa S. Jann K.. (2013). EEG microstates associated with salience and frontoparietal networks in frontotemporal dementia, schizophrenia and Alzheimer's disease. Clin. Neurophysiol. 124, 1106–1114. 10.1016/j.clinph.2013.01.005 23403263

[B30] Pascual-Marqui R. Michel C. Lehmann D. (1995). Segmentation of brain electrical activity into microstates: model estimation and validation. IEEE Trans. Biomed. Eng. 42, 658–665. 10.1109/10.391164 7622149

[B31] Peinado A. B. Alfaro-Magallanes V. M. Romero-Parra N. Barba-Moreno L. Rael B. Maestre-Cascales C.. (2021). Methodological approach of the iron and muscular damage: female metabolism and menstrual cycle during exercise project (IronFEMME Study). Int. J. Environ. Res. Public Health 18:735. 10.3390/ijerph18020735 33561085 PMC7831010

[B32] Poulsen A. T. Pedroni A. Langer N. Hansen L. K. (2018). Microstate EEGlab toolbox: an introductory guide. bioRxiv. 10.1101/289850

[B33] Strahler J. Hermann A. Schmidt N. Stark R. Hennig J. Munk A. (2020). Food cue-elicited brain potentials change throughout menstrual cycle: modulation by eating styles, negative affect, and premenstrual complaints. Hormones Behav. 124:104811. 10.1016/j.yhbeh.2020.104811 32592725

[B34] Thatcher R. W. Walker R. A. Biver C. J. North D. N. Curtin R. (2003). Quantitative EEG normative databases: validation and clinical correlation. J. Neurother. 7, 87–121. 10.1300/J184v07n03_05

[B35] Virtanen P. Gommers R. Oliphant T. E. Haberland M. Reddy T. Cournapeau D.. (2020). SciPy 1.0: fundamental algorithms for scientific computing in Python. Nature Methods 17, 261–272. 10.1038/s41592-020-0772-5 32015543 PMC7056644

[B36] Zheng K. Wang H. Hou B. You H. Yuan J. Luo K.. (2017). Malnutrition-inflammation is a risk factor for cerebral small vessel diseases and cognitive decline in peritoneal dialysis patients: a cross-sectional observational study. BMC Nephrol. 18:366. 10.1186/s12882-017-0777-1 29262796 PMC5738894

